# Detoxification of comfrey (*Symphytum officinale* L.) extract using natural deep eutectic solvent (NADES) and evaluation of its anti-inflammatory, antioxidant, and hepatoprotective properties

**DOI:** 10.3389/fphar.2023.1012716

**Published:** 2023-03-03

**Authors:** Andiri Niza Syarifah, Herman Suryadi, Hayun Hayun, Adelina Simamora, Abdul Mun’im

**Affiliations:** ^1^ Graduate Program, Universitas Indonesia, Faculty of Pharmacy, Depok, Indonesia; ^2^ Department of Biology Pharmacy, Faculty of Medicine, Universitas Pembangunan Nasional Veteran Jakarta, Jakarta, Indonesia; ^3^ Laboratory of Microbiology and Biotechnology, Faculty of Pharmacy, Universitas Indonesia, Depok, Indonesia; ^4^ Laboratory of Pharmaceutical, Medicinal and Bioanalysis, Faculty of Pharmacy, Universitas Indonesia, Depok, Indonesia; ^5^ Department of Biochemistry, Faculty of Medicine and Health Sciences, Krida Wacana Christian University, Jakarta, Indonesia; ^6^ National Metabolomic Collaborative Research Center, Faculty of Pharmacy, Universitas Indonesia, Depok, Indonesia; ^7^ Department of Pharmacognosy-Phytochemistry, Faculty of Pharmacy, Universitas Indonesia, Depok, Indonesia

**Keywords:** Symphytum officinale, comfrey, rosmarinic acid, lycopsamine, NADES, HPLC-DAD, detoxification, bioactivities

## Abstract

Comfrey (*Symphytum officinale* L.) contains rosmarinic acid which has different pharmacological activities, such as antioxidant and anti-inflammatory activities. However, the medicinal use of comfrey is limited by the hepatotoxic effect of lycopsamine in comfrey, which overshadows the health benefits of rosmarinic acid. Natural deep eutectic solvents (NADES) have a wide range of extraction properties, that provides a new approach to the detoxification of comfrey. In the present study, betaine-based and choline chloride-based NADES were screened for selective extraction of rosmarinic acid over lycopsamine. Ultrasonication was used in conjunction with NADES extraction. The chemical profile of the NADES extracts on antioxidant, anti-inflammatory and hepatotoxic activities were investigated using some chemical reagents. Betaine-urea (1:2 molar ratio, 50% water) obtained the highest content of rosmarinic acid and a low level of lycopsamine (1.934 and 0.018 mg/g, respectively). Betaine-urea was also shown to be more effective to extract rosmarinic acid compared to methanol-UAE under the same conditions, which gave lower rosmarinic acid and higher lycopsamine levels (0.007 and 0.031 mg/g, respectively). Betaine-urea extracts showed higher antioxidant and anti-inflammatory properties as compared with other NADES extracts, however, had lower hepatotoxic profile. This study recommends the use of betaine-urea to detroxify comfrey to open wider opportunities for the development of comfrey for medicinal use.

## 1 Introduction

Comfrey (*Symphytum officinale* L*.,* Boraginaceae) (Wilkinson, 2003), is a perennial plant with long rough leaves and white, creamy yellow, or pale purple flowers. The leaves and roots are often consumed (Frost et al., 2013). Comfrey is a well-known traditional plant medicine with a long therapeutic history. Its preparations have been widely used for the treatment of severe muscle and joint problems, wounds and bone fractures, and inflammation (Seigner et al., 2019). Traditionally, comfrey is boiled as tea to help treat gastritis, ulcers, and lung disorders (Wilkinson, 2003). Comfrey is also used as an external preparation to heal, ulcers, sprains, and fractured bones (Smith and Jacobson, 2011). Comfrey can survive in tropical climates, thus is widespread in Indonesia (Kementerian Kesehatan Republik Indonesia, 2017), where people use to reduce inflammation, aches and pains, diarrhea, typhoid, heartburn, and bone fractures (Kinho et al., 2011).

**FIGURE 1:**
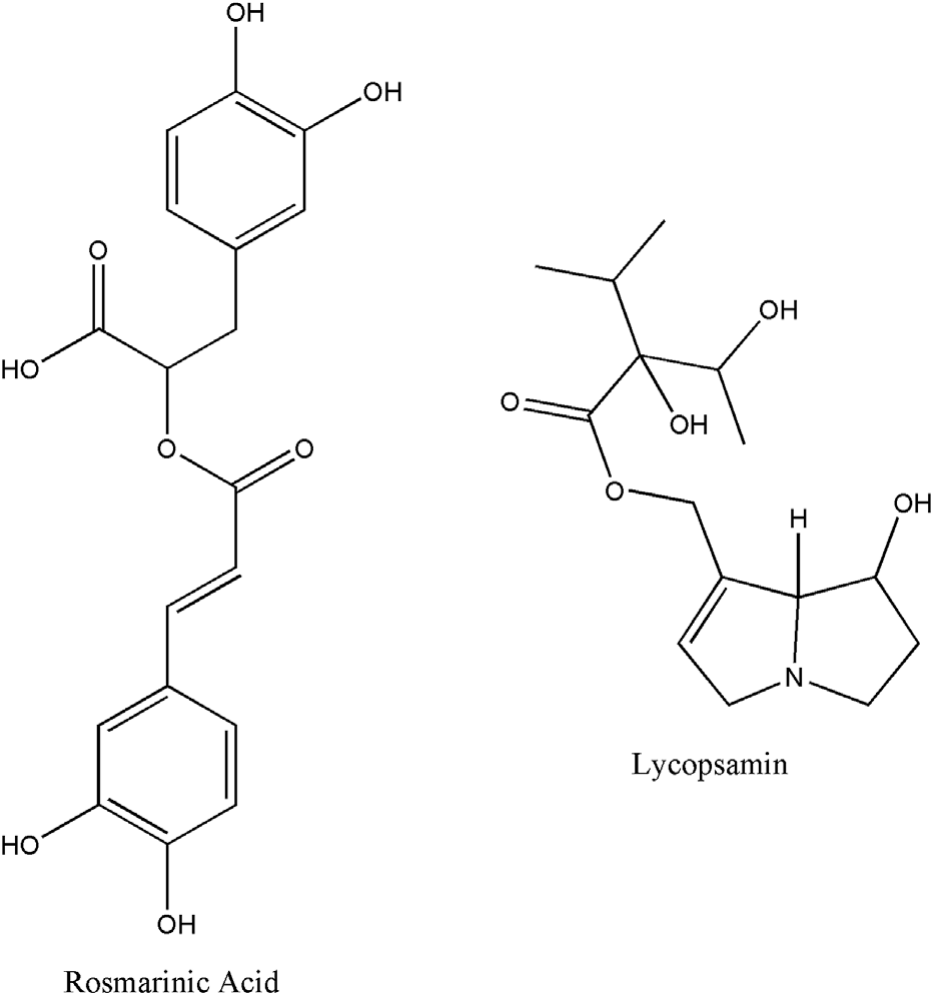
The chemical structure of rosmarinic acid and lycopsamine.

Rosmarinic acid, which is the caffeic acid ester of 3,4-dihydroxyphenyllactic acid (Tawfeeq et al., 2018), is a common phenolic acid in comfrey (Trifan et al., 2018). Shoots and leaves of comfrey contained the most of rosmarinic acid (Dresler et al., 2017). Previous studies showed some biological activities of rosmarinic acid and its derivates such as anti-inflammatory, antioxidant, antibacterial, anti-hyperglycemic and anti-allergic activities (Tawfeeq et al., 2018; Salehi et al., 2019). The chemical structure of rosmarinic acid is shown in Figure 1.

Nevertheless, there are some restrictions for comfrey applications. Comfrey has been linked to liver damage (Frost et al., 2014), which is associated with the presence of pyrrolizidine alkaloids in the plant. Pyrrolizidine alkaloids such as lycopsamine are significantly found in of the comfrey plant (0.2%–0.4%) (Liu et al., 2009). It has been reported that lycopsamine was responsible for the hepatotoxic effects (Zsuzsanna et al., 2015; Moreira et al., 2018; Salehi et al., 2019). This is probably due to its metabolism by CYP3A4 into pyrrole derivatives, which are hepatotoxic and pulmotoxic (Janes et al., 2012). The lycopsamine-induced hepatotoxicity obscures the advantages of rosmarinic acid in comfrey. Therefore, improvement of comfrey extraction is required to maximize rosmarinic acid levels while minimize lycopsamine level in order to produce the best therapeutic effect. However, selective extraction of rosmarinic acid over lycopsamine is a challenging task due to the substantial presence of lycopsamine in comfrey. Chemical structure of lycopsamine showed in Figure 1.

Natural Deep Eutectic Solvents (NADES) have emerged as a green approach for maximizing the potential and advantages of natural metabolites (Zhang et al., 2018). NADES are made of natural components, that can be found in cells and organisms, such as organic acids, urea, glycerol, and sugars. NADES are considered safe due to non-volatility and environmentally friendly compared to organic solvents which are flammable and non-biodegradable (Cao et al., 2020). The inclusion of hydrogen bond donors (HBD) and hydrogen bond acceptors in NADES form strong hydrogen bond interactions between them, leading to low melting point mixtures. In addition, NADES have some desirable properties including ease of preparation, good extraction ability, and low cost (Cysewski and Jeliński, 2019; Liu et al., 2019). NADES can also improve the stability and shelf life of metabolites in extracts (Obluchinskaya et al., 2021). Furthermore, the solubility of metabolites dissolved in NADES has been observed to increase (Cao et al., 2020). NADES have been reported to successfully extract some natural metabolites such as phenolic metabolites in *Rosmarinus officinalis* L. and *Mentha x piperita* L. (Jeong et al., 2018; Barbieri et al., 2020) as well as alkaloid metabolites in *Crinum x powelii* Baker bulbs and *Coffea canephora* Pierre ex A.Froehner (Shawky et al., 2018; Syakfanaya et al., 2019). The present work aims to develop green approach for selective extraction of rosmarinic acid over lycopsamine from the leaves of comfrey (*Symphytum officinale* L). Betaine- and choline chloride-based NADES were formed with glycerol, urea, and sucrose. NADES extracts were analyzed by an HPLC-DAD. Further, the antioxidant, anti-inflammatory, and hepatotoxic chemical profiles of NADES extracts were compared with those of conventional solvent.

## 2 Materials and methods

### 2.1 Materials and chemical reagents

The following materials were utilized in this study. Comfrey (*Symphytum officinale* L.) leaves were purchased from local farmer in Karanganyar, Indonesia and authenticated in The Faculty of Biology, Universitas Gajah Mada. Choline chloride, Urea, 2,2-diphenyl-1-picrylhydrazyl (DPPH) and 2,2′-azino-bis (3-ethylbenzothiazoline-6-sulfonic acid) (ABTS) were purchased from Sigma-Aldrich (St. Louis, MO, United States). Glycerol was obtained from PT Molex Ayus (Jakarta, Indonesia). Sucrose was obtained from PT Brataco (Jakarta, Indonesia). Betaine was bought from Shandong Ruihong Biotech (Jinan city, China). Standards of lycopsamine and rosmarinic acid were bought from Phytolab (Vestenbergsgreuth, Germany). Vitamin C and sodium diclofenac were purchased from National Food and Drug Agency of Indonesia (Jakarta, Indonesia). Bovine serum albumin (BSA) was purchased from Himedia (Maharashtra, India). Solvents used were of HPLC grade.

### 2.2 NADES preparation

NADES were synthesized by heating and stirring method as described elsewhere (Dai et al., 2013). A hydrogen bond donor (HBD) and a hydrogen bond acceptor (HBA) component in a specific molar ratio were added into a beaker glass. The mixture was stirred and heated at 80°C until a clear solution was generated. All compounds used in the preparation of NADES are readily accessible and low-cost, Table 1.

**TABLE 1:** NADES preparation for extraction.

NADES components		Molar ratio	Appearance
Component 1 (HBA)	Component 2 (HBD)		
Choline chloride	Glycerol	1:2	Transparent colorless liquid
	Urea	1:2	Transparent colorless liquid
	Sucrose	1:2	Light-yellow viscous liquid
Betain	Glycerol	1:2	Transparent colorless liquid
	Urea	1:2	Transparent colorless liquid
	Sucrose	1:2	Light-yellow viscous liquid

### 2.3 Ultrasound-assisted extraction of comfrey with NADES

All NADES extractions were carried out using an ultrasonic bath (Krisbow, Jakarta, Indonesia). Comfrey leaf powder was placed in a flask and blended with NADES in a solid to liquid ratio of 1:20. Ultrasonication-assisted extraction (UAE) was carried out for 45 min at 50°C (35 W, 42 kHz). The solid residue was separated from the liquid phase by centrifugation (Hettich Zentrifugen, Germany), at 4,500 rpm for 17 min. The supernatant was filtered through a 0.45 µm Whatman micropore filter paper. The filtrate was collected and kept at 4°C until further analysis. The filtrate was collected and kept in a refrigerator until further examination (Duan et al., 2016).

To get comparative data, methanol extraction in conjunction with UAE was performed. The methanol-UAE extraction was conducted for 50 min at 50°C with a ratio of solid to liquid of 1:60. The filtrates were evaporated using a rotary vacuum evaporator (R-215, Buchi, Switzerland). The concentrated filtrates were collected and refrigerated for future use in analysis (Janes et al., 2012).

### 2.4 Quantification of rosmarinic acid and lycopsamine metabolites in comfrey extract

The contents of rosmarinic acid and lycopsamine in comfrey extracts were determined by an Agilent 1200 HPLC system (Analytical Instruments, CA, United States) with a UV-Vis spectroscopy detector. A Zorbax Eclipse XDB-C18 column (4,6 × 150 mm, 5 μm; Agilent Technologies Co., Ltd, Santa Clara, United States) was used for chromatographic separation. The investigated substances were separated using isocratic elution for 15 min with 30% of acetonitrile as phase B and 0.1% of formic acid as phase A. The injection volume was 20 µL. Column separation was carried out at room temperature with a mobile phase at flow rate of 0.5 mL/min. The HPLC chromatogram of rosmarinic acid and lycopsamine was identified at 210 and 330 nm, respectively (Syarifah et al., 2022). Calibration curves were generated for rosmarinic acid (4–20 μg/mL) and lycopsamine (10–100 ng/mL) at five concentration points.

### 2.5 Antioxidant profile of the NADES preparation

#### 2.5.1 DPPH radical scavenging profiling

DPPH radical scavenging profiling was conducted based on a reported method (Vongsak et al., 2018). In a 96 well plate was mixed 100 µL of comfrey extract at different concentrations in methanol (4–20 μg/mL) and 100 µL of 253.6 µM of DPPH reagents in methanol. The plate was incubated for 20 min at 37°C, thereafter the absorbance was measured at 560 nm on a microplate reader (Glomax Promega, United States). Methanol in place of sample was used as a blank. Rosmarinic acid, lycopsamine, and vitamin C were used as positive control. All measurements were conducted in triplicate. The DPPH chemical profile was determined using Equation 1:

$$Chemical\;Profile\;of\;Inhibition\;\left(\%\right)=100\;\%\times \frac{A_{blank}-A_{sample}}{A_{blank}}$$ (1)

Where A_blank_ was absorbance of the blank solution and A_sample_ was the absorbance of sample solution. The IC_50_ values were calculated by a linier regression generated from the inhibition percentage plotted against concentration.

#### 2.5.2 ABTS radical scavenging profiling

ABTS•+ cation radical reagents were generated by mixing equal volumes of 7 mM ABTS reagent and 2.45 mM potassium persulfate solutions in the dark at room temperature for 16–20 h. ABTS•+ solution was then adjusted to an absorbance of 0.70 ± 0.02 at 600 nm by adding methanol. Into a 96-well plate was added 100 µL of comfrey extracts at different concentrations and 100 µL of ABTS•+ solution. The reduction in absorbance was observed after 6 min (Trifan et al., 2018). Experiments were conducted in triplicate. The ABTS•+ radical scavenging profiling was calculated using Eq. 1.

### 2.6 Anti-inflammatory profile of the NADES preparation

Anti-inflammatory profile of comfrey extracts was evaluated using reagent based on their inhibition on protein denaturation, according to a method described elsewhere (Heendeniya et al., 2018). Reaction mixture was prepared containing 100 µL of samples at different concentrations (4–20 μg/mL), 140 µL of freshly prepared phosphate buffer saline (10 mM, pH 6.3), and 20 µL of egg albumin extracted from hen egg or 45 µL of bovine serum albumin solution (0.5%, w/v). The reaction mixture was incubated at 37°C for 15 min. The mixture was further heated at 70°C for 5 min. After cooling down at ambient temperature, the turbidity of the reaction mixture was measured at 600 nm using a microplate reader (Glomax Promega, United States) against phosphate buffer saline as a blank. Sodium diclofenac was used as a positive control. The experiments were performed in triplicate. The inhibitory profile was determined based on Eq. 1.

### 2.7 Hepatotoxic profile of the NADES preparation

#### 2.7.1 Cells culture conditions and treatment

Human hepatocytes (HepaRG) were obtained from Sigma-Aldrich (United States). HepaRG were cultured in Roswell Park Memorial Institute (RPMI) 1,640 medium, supplemented with 10% (v/v) fetal bovine serum (FBS), 1% antibiotics, and 1% fungizone. The cells were maintained at 37°C with 5% CO_2_. Trypsin (0.25%) was used to harvest cells at 90%–100% confluency. Trypan blue was used to color the cells for death cell detection. Comfrey stock solution (1,000 g/mL) was prepared in DMSO, which was further diluted with basal media to obtain reagen solutions of different concentrations (You et al., 2018).

#### 2.7.2 Cell viability assay

The cytotoxic effect of comfrey on HepaRG cells was assessed using the MTS 3-(4,5-dimethylthiazol-2-yl)-5-(3-carboxymethoxyphenyl)-2-(4-sulfophenyl)-2H-tetrazolium) reagent. The cells were seeded in a 96-well plate at a density of 20,000 cells/well with culture medium (FBS, 10%, v/v). The next day, the cells were treated with various concentrations of comfrey extracts 0, 25, 50 and 100 g/mL. After incubation for 24 h, 10 µL of MTS solution was added. The mixtures were further incubated for 1–4 h at 37°C. The absorbance was measured with an ELISA reader at 490 nm. DMSO solution (0.1%) was used as a negative control. The LC_50_ values were calculated using the probit model (You et al., 2018).

$$Chemical\;Profile\;of\;Cell\;Death\;\left(\%\right)=100\;\%\times \frac{A_{blank}-A_{sample}}{A_{blank}}$$ (2)

The LC_50_ values were calculated by a linier regression generated from the chemical profile of cell death percentage plotted against concentration.

### 2.8 Fourier-transform infrared spectroscopy (FTIR) assay

Sample in FTIR assay was 10 mg of rosmarinic acid standard, 10 mL of NADES betain-urea liquid and 10 mL of 1% mixture of rosmarinic acid standard in NADES betain-urea. The FTIR test was carried out using the Thermo Scientific - Nicolet iS50 FTIR + NIR Spectrometer provided by the ILRC laboratory of the University of Indonesia. The FTIR spectra was observed structural changes of rosmarinic acid due to solvation in NADES.

### 2.9 Proton- nuclear magnetic resonance (^1^H-NMR) assay

Sample in ^1^H-NMR assay was 10 mg of rosmarinic acid standard, 10 mL of NADES betain-urea liquid and 10 mL of 10% mixture of rosmarinic acid standard in NADES betain-urea. ^1^H-NMR assay was carried out using the JEOL-ECZ 500R with CD3OD as solvent at LIPI Serpong. The ^1^H-NMR assay spectra were evaluated the hydrogen bond of sample.

### 2.10 Morphology assay

Morphological observation was carried out by a Jeol JSM-IT200 equipment. Dried plant materials (leaf powder, methanol, and NADES extracts) each 50 g were aerated to dryness. Samples were further dehydrated under high vacuum, then coated with thin layer of gold. An accelerated voltage of 20 kV was used. Samples were observed under SEM analyser for the shape and the stomatas.

## 3 Results

In this work, six different combinations of NADES were screened for selective extraction of rosmarinic acid over lycopsamine from comfrey leaves (Table 1). The studied NADES were composed of choline chloride or betaine as hydrogen bond acceptor (HBA), whereas urea, sucrose, or glycerol were used as hydrogen bond donor (HBD), in a 1:2 molar ratio of HBA:HBD. Each NADES was added with 50% (v/v) water to reduce the viscosity.

**FIGURE 2:**
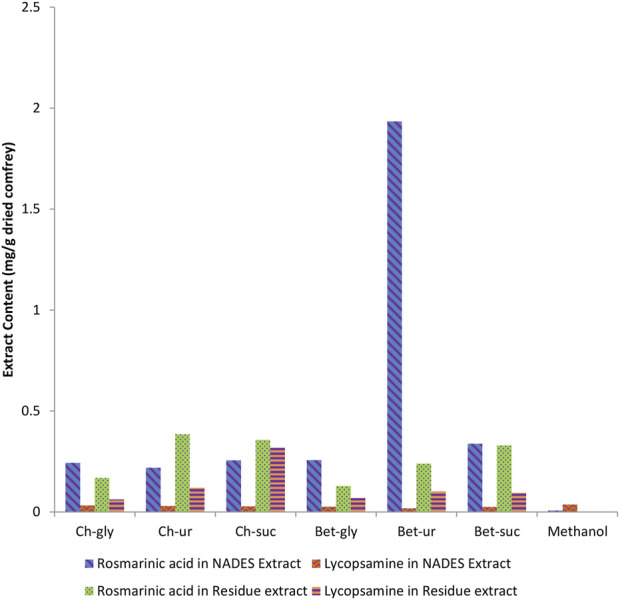
Comparison of rosmarinic acid and lycopsamine contents obtained by extraction of comfrey leaves in different NADES. The residue was further re-extracted using methanol to assess the contents of the bioactive metabolites.

Ultrasonication was used to assist extraction. The same basic extraction conditions were followed (50°C, 1:20 solid to liquid ratio, and 50 min extraction time). The residue of NADES extracts was re-extracted using methanol to evaluate the effectiveness of the extraction process. Methanol extraction using the same extraction procedures was used for comparison purpose. Figure 2 shows the metabolite of rosmarinic acid and lycopsamine in each NADES following extraction of comfrey leaves.

### 3.1 Quantification of rosmarinic acid and lycopsamine

**FIGURE 3:**
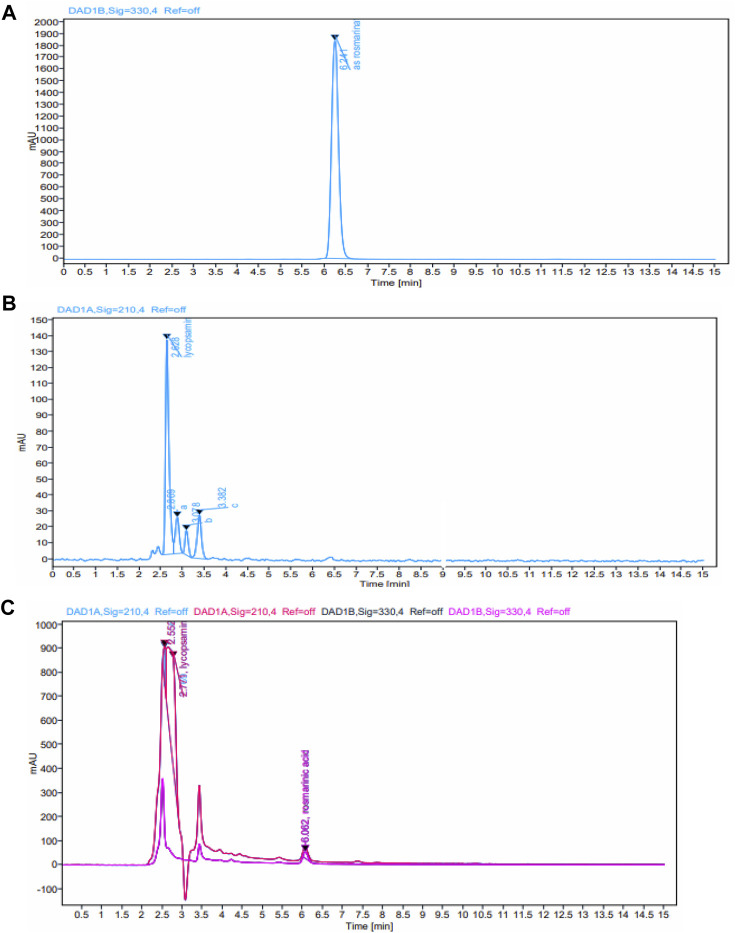
HPLC chromatograms of the standard metabolites **(A)** rosmarinic acid **(B)** lycopsamine, and **(C)** extracts obtained by ultrasound assisted extraction of comfrey leaves.

HPLC method was used for analyses of rosmarinic acid and lycopsamine contents in the extract. The chromatogram of standard metabolites is shown in Figure 3. Analyses results in Figure 2 shows that each NADES gave different levels of rosmarinic acid and lycopsamine. However, all NADES showed better extraction performance with regard to rosmarinic acid and lycopsamine contents, when compared with methanol extraction. Of these NADES, betaine-urea extracted the highest level of rosmarinic acid (1.934 mg/g), while successfully maintain the lowest content of lycopsamine (0.018 mg/g). Significant amount of lycopsamine and low amount of rosmarinic acid was quantified from the residue which was re-extracted using methanol (0.102 and 0.240 mg/g, respectively). It was noted that, the least amount of rosmarinic acid was quantified from methanol extract (0.007 mg/g).

### 3.2 Antioxidant profile of comfrey extract

**FIGURE 4:**
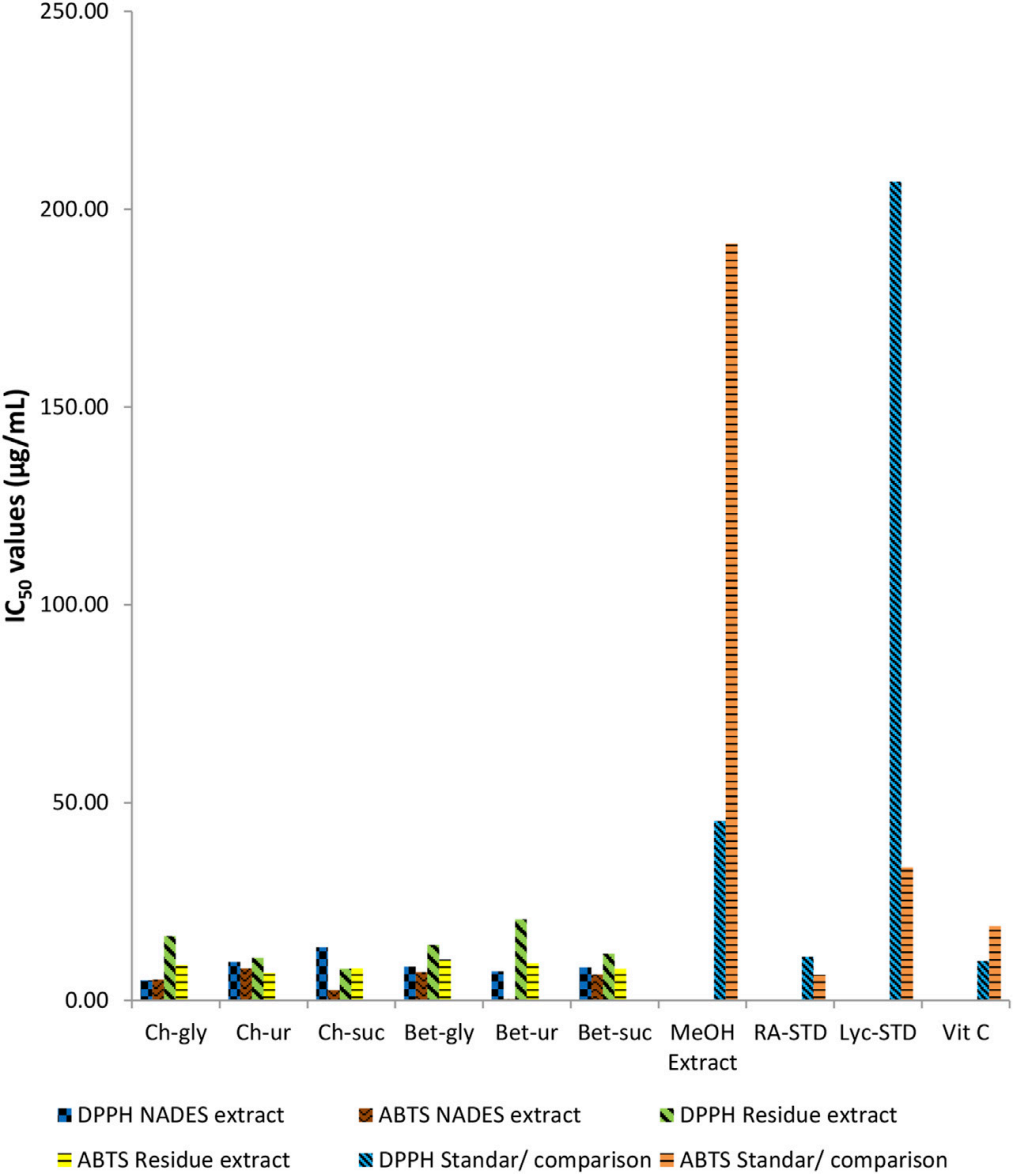
Comparison of antioxidant activities of comfrey leaf extracts by different NADES and methanol, and those of standard metabolites.

The chemical antioxidant profile of comfrey extracted by different NADES are shown in Figure 4. For comparison, the antioxidant profile of methanol extract of comfrey and standards were also determined. In general, all NADES extracts showed significantly higher antioxidant profile by DPPH and ABTS assays when compared with methanol extract. It was also worth noting that the antioxidant profile of NADES extracts were comparable to those of standards rosmarinic acid, lycopsamine, vitamin C in both assays. Comfrey extracted by betaine-urea showed the highest ABTS scavenging chemical profile (IC_50_ 0.33 μg/mL), whereas its chemical profile by DPPH reagent was the second strongest (IC_50_ 7.36 μg/mL) after extract derived from choline chloride-glycerol.

### 3.3 Anti-inflammatory profile of comfrey extract

**FIGURE 5:**
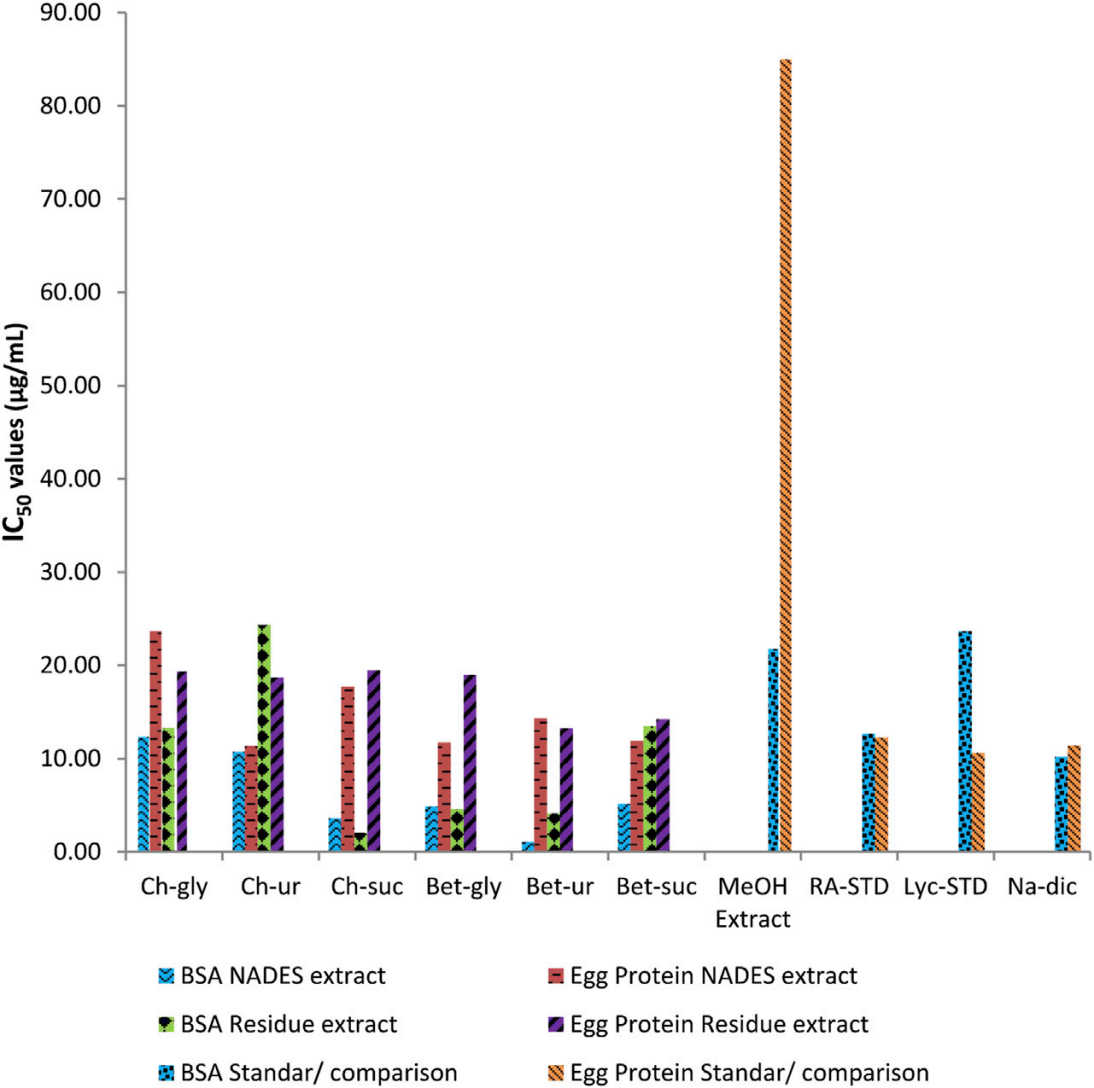
Comparison of anti-inflammatory activities of comfrey leaf extracts by different NADES and methanol, and those of standard metabolites.

The ability of NADES extracts to profile inflammation was evaluated chemically by protein denaturation inhibition assay, using BSA and egg protein. Results are shown in Figure 5. Generally, all NADES extracts exhibited stronger anti-inflammatory chemical profile compared to methanol extract. Some of NADES extracts showed even stronger chemical profile when compared to standards rosmarinic acid, lycopsamine, and sodium diclofenac. Compared to other NADES extracts, betaine-urea extract was the most active to inflammation profile using BSA, as shown by the lowest IC_50_ value. Strong profile was also shown when using egg protein, although other NADES extracts showed stronger profiles, such as choline chloride-urea, betaine-glycerol, and betaine-sucrose extracts.

### 3.4 Hepatotoxic profile

The LC_50_ values of hepatotoxic profile of extracts are shown in Table 2. NADES betain-urea extract showed the highest LC_50_ of cell death using chemical reagent. In contrast, the residue obtained from betaine-urea extract exhibited the lowest LC_50_. The methanol extract showed significant hepatotoxicity profile as indicated by a low LC_50_.

**TABLE 2:** Hepatotoxic profiling of extract.

Sample	LC_50_ (g/kg)
NADES extract of Choline chloride-urea	90.64
NADES extract of Betain-urea	800.00
Methanol extract	36.90
Residue extract of Choline chloride-urea	115.32
Residue extract of Betain-urea	7.91

## 4. Discussion

### 4.1 Quantification of rosmarinic acid and lycopsamine

The present study compared betaine based- and choline chloride-based- NADES to achieve high rosmarinic acid and low lycopsamine extraction. Rosmarinic acid (a phenolic metabolites) was commonly found in the Lamiaceae and Boraginaceae families (Öztürk et al., 2011), including in comfrey. Together with rosmarinic acid, lycopsamine was also present in significance in comfrey. Lycopsamin was the most abundant PA found in the leaves and in this study the leaves of the komfrey plant were only used. Lycopsamine as one of the most widely used lycopsamines from leaf parts is expected to represent a significant reduction in PA from extracting leaf parts of comfrey plants (Salehi et al., 2019). The medicinal use of comfrey is limited by the hepatoxic effect of lycopsamine (Cao et al., 2008). Selective extraction was achieved by using betaine-urea, which was able to extract rosmarinic acid more than eight times higher than choline chloride-urea. Betaine-urea was also able to selectively target rosmarinic acid over lycopsamine compared to other studied NADES (Figure 2). The re-extraction of the residue identified decreased level of rosmarinic acid, whereas observed high content of lycopsamine. As seen in Figure 2 lycopsamine was shown to be better extracted using methanol. These results demonstrated successful selectivity of betaine-urea extraction towards rosmarinic acid, indicating potential function to detroxify comfrey. As previously mentioned, despite the different health benefits of comfrey, its medicinal use is limited by the hepatoxic effect of lycopsamine (Cao et al., 2008), which was found in significance in comfrey.

NADES constituents (HBA and HBD) and their molar ratio determine the physicochemical properties of NADES, which influence the affinity between target metabolites and the extraction solvents (Table 1). The NADES component forms hydrogen bond interactions and Van der Walls interactions by pairing a hydrogen bond acceptor (HBA) and a hydrogen bond donor (HBD) at suitable molar ratios (Smith et al., 2014; Kalhor and Ghandi, 2019). However, the formation of hydrogen bond networks in NADES leads to highly viscous solvents. Thus, in the present study, each NADES was added with water (50%, v/v) which greatly reduced the viscosity. Excessive water addition (more than 50%) is avoided as this addition destroys hydrogen bond interactions between HBA and HBD (Dai et al., 2013). The incorporation of water in NADES may also modulate the polarity of NADES. These effects subsequently increased mass transfer and enhanced extraction efficiency.

Comfrey leaves showed the morphology of the dried comfrey leaf cells before extraction which was still in a large state after extraction in methanol showed the morphology of comfrey leaves after extraction with methanol and sonication, the damage to the leaf cells became smaller but the stomata part of the sample was still intact. Meanwhile, samples of NADES extract experienced greater stomata damage and this was in line with high levels because the more damaged the stomata, the higher the metabolites that came out into the solvent.

**FIGURE 6:**
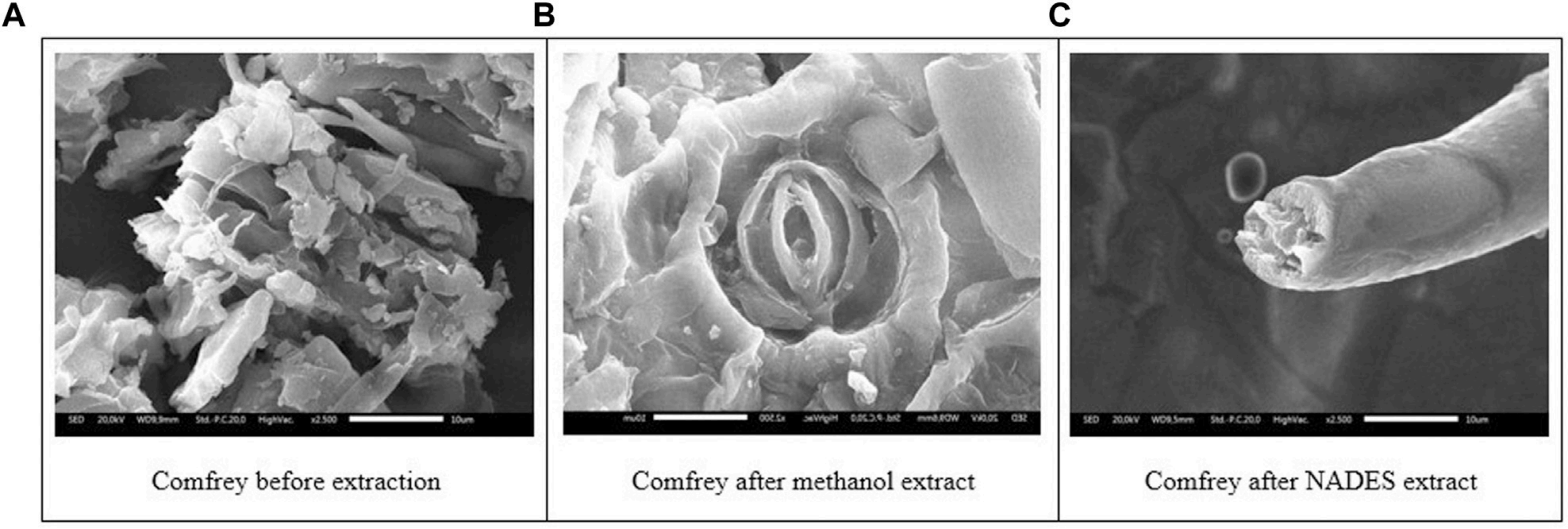
Scanning electron micrographs of comfrey leaves before extraction **(A)**, after extraction in methanol **(B)**, and NADES **(C)**.

In the present work, ultrasonication was employed as the energy source for extraction. The effect of NADES combined with ultrasonication can be observed on the morphological changes of the comfrey leaves as seen in the SEM images (Figure 6). The cavitation bubbles implode on the cell surface and disrupt the cell wall, allowing the particle size to decrease and the mass transfer process from the cell into the solvent to rise. Figure 6B shows that ultrasonication in methanol extraction broke down and destroyed the morphological structure of comfrey (Figure 6B). This helps release various metabolites into the solvent during the extraction process. The UAE-NADES extraction was seen to change the leaf surface to become large and formed a fibrous network (Figure 6C). This may be due to the implosion cavitation bubbles near the leaf cell surface that may erode the leaf structure and help release metabolites into the solvent. The ultrasound-assisted extraction (UAE) method yielded superior secondary metabolite extraction results than the NADES solvent with the maceration technique (Mansinhos et al., 2021). Therefore, UAE extraction procedures were performed for both methanol and NADES solvents (Kanedi et al., 2017; Ruesgas-Ramón et al., 2017; Vinatoru et al., 2017).

**FIGURE 7:**
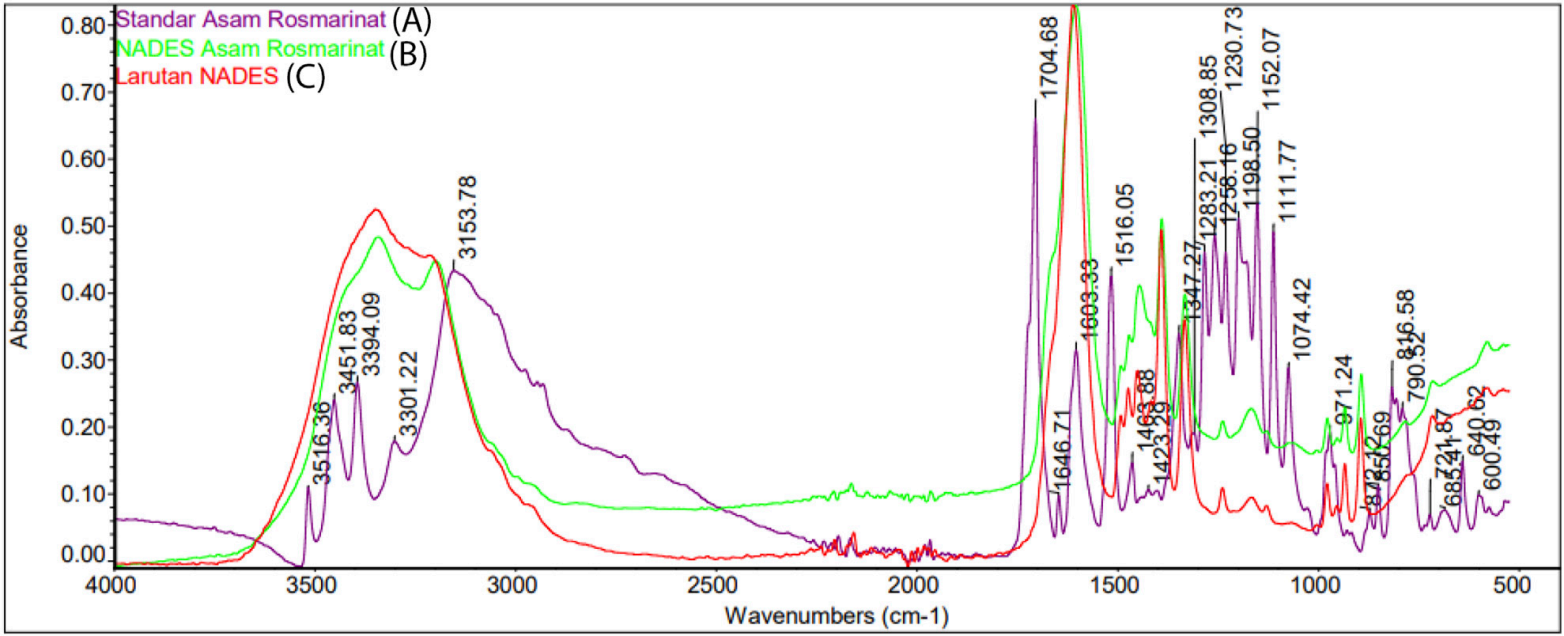
FTIR spectra of **(A)** standard rosmarinic acid **(B)** rosmarinic acid in NADES (betaine-urea), and **(C)** NADES (betaine-urea).

**FIGURE 8:**
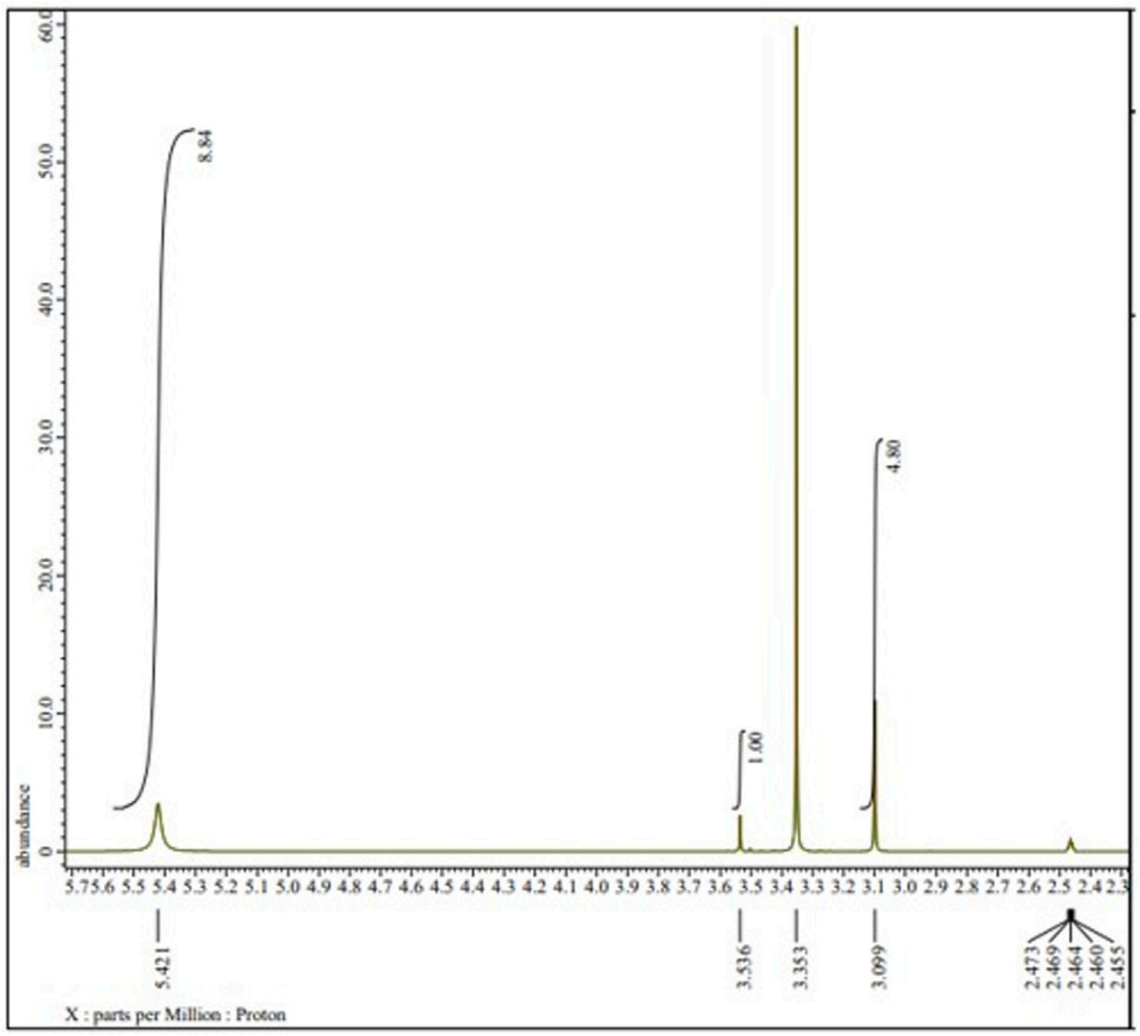
^1^H-NMR of rosmarinic acid in betaine-urea.

**FIGURE 9:**
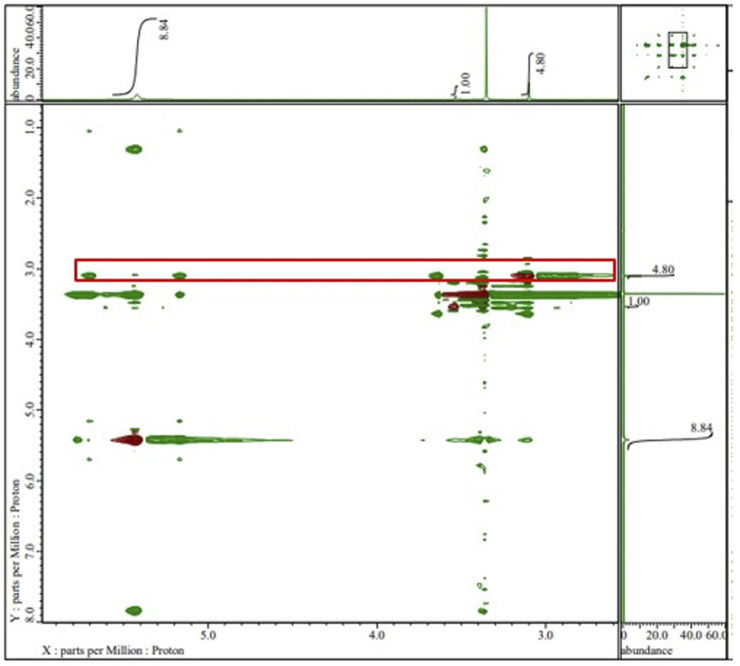
NOESY spectra of rosmarinic acid in betaine-urea at 298 K.

The FTIR absorption spectrum was used to observed structural changes of rosmarinic acid due to solvation in NADES (Figure 7). For this purpose, The FTIR spectra of rosmarinic acid, NADES (betaine-urea), and rosmarinic acid in NADES were compared. FTIR spectra of standard rosmarinic acid in NADES and NADES (betaine-urea) showed characteristic hydroxyl peaks in the 3,000–3,500 cm^−1^ region. In addition, the solubility of rosmarinic acid in NADES solution was seen at a low intensity peaks in the 2,500–3,000 cm^−1^ region. The peaks in the 1,000 cm^−1^ region indicated the hydrogen bonds formed between the hydroxyl and carbonyl groups. This shows that hydrogen bonding plays an important role in the high dissolution of phytochemical metabolites in NADES. The displacement of OH group signals in ^1^H NMR proton spectra confirmed the establishment of hydrogen bonds, as shown in Figure 8. In the ^1^H–^1^H NOESY spectra (Figure 9), it was observed the presence of the spatial heteromolecular interaction between water molecules and the OH groups of the NADES components, supporting the establishment of hydrogen bonds between them.

Previously, choline chloride and betaine-based NADES have been used for the extraction of phenolic metabolites and alkaloids from plant materials. For example, phenolic metabolites extracted from *Rosmarinus officinale* L. using choline chloride-1,2 propanediol (1:2 molar ratio, 10% of water content) gave the best results in rosmarinic acid extraction (13,563.0 μg/g) (Barbieri et al., 2020). Extraction of caffeine components (alkaloid derivatives) in *Camellia sinensis* L. Kuntze gave the best results using NADES-UAE choline chloride-lactic acid (1:1 molar ratio, 31% water content) (Cai et al., 2019). Betaine-urea NADES was used to extract chlorogenic acid (a phenolic metabolites) and caffeine (an alkaloid) from *Coffea canephora* Pierre ex A.Froehner, yielding higher chlorogenic acid (28.62 mg/g) and lower caffeine (7.89 mg/g) (Syakfanaya et al., 2019). In the present study, betaine-urea was proved to be selective toward rosmarinic acid but not lycopsamine. These results are indicative of prospective use of betaine-urea to detoxify comfrey due to the lower content lycopsamine in the betaine-urea extract. This finding may lead to the more usability of comfrey for its antioxidant and anti-inflammatory chemical profiles, whereas at the same time less toxic to liver.

### 4.2 Antioxidant profile of comfrey extract

In the current study, antioxidant profile of NADES extracts were carried out to investigate the efficiency of extraction. The antioxidant profiles were evaluated based on their scavenging ability on the radical DPPH and ABTS reagent molecules. Both DPPH and ABTS radicals have sufficient capacity to accept electrons and hydrogen atoms from samples containing antioxidant metabolites (Francenia Santos-Sánchez et al., 2019).

Previously, comfrey roots extracted by aqueous ethanol were demonstrated to have high antioxidant activities as measured by DPPH radical reagent and ABTS cation radical reagent scavenging abilities, reducing power, and 15-lipoxygenase inhibition assay, which were associated with the phenolic metabolites in comfrey root extract (Trifan et al., 2018). Similarly, Neagu et al. (2010) found correlation between the antioxidant capacity profiled using DPPH and ABTS reagents and polyphenol content of the root ethanolic extracts (Neagu et al., 2010). It was well known that extraction capacity of NADES towards phenolic metabolites were closely correlated with the antioxidant profile of the extracts (Fu et al., 2021). Rosmarinic acid which was the target metabolites in this study belongs to the category of phenolic metabolites. Similar findings were observed in the present study. Betaine–urea extract, which was demonstrated to obtain the highest content of rosmarinic acid (Figure 2), gave the highest antioxidant profile, when compared with other NADES and methanol extracts (Figure 3). These results indicate that rosmarinic acid may be responsible for the antioxidant chemical profile observed in the NADES extracts. Rosmarinic acid contains two phenolic rings, both of which have ortho-hydroxy groups which may be crucial in its antioxidant activity. The selective extraction towards rosmarinic acid was further confirmed by antioxidant profile of residues of NADES extracts. It was expected that betaine-urea extract showed the weakest antioxidant profile. This is due to the lowest content of rosmarinic acid left in the comfrey residue. Rosmarinic acid was reported to have therapeutic effects due to its antioxidant activity (Jurić et al., 2021), (Benedec et al., 2015). In addition, rosmarinic acid has been reported to have different biological activities such as HIV1, antitumor, antihepatitic suppression, liver protection, blood clot suppression, and anti-inflammatory (Aldoghachi et al., 2021).

### 4.3 Anti-inflammatory profile of comfrey extract

Endogenous variables that promote the generation of reactive oxygen species can initiate the inflammatory process. If free radicals are present in significant concentrations in cells, they will cause tissue damage (Francenia Santos-Sánchez et al., 2019). The presence of protein denaturation events is associated to the inflammatory response. Protein denaturation in cells or intercellular molecules linked to tissue injury As a result, a metabolites’s capacity to suppress protein denaturation indicates possible anti-inflammatory effect (Osman et al., 2016). In the present study, comfrey leaf extracts were observed to inhibit protein denaturation (Figure 5). The comfrey plant has long been used as a topical analgesic and anti-inflammatory. Comfrey plants can be used to alleviate muscular discomfort, mend fractures and wounds, and function as an anti-inflammatory (Seigner et al., 2019). Previous research has indicated that comfrey plants have analgesic and anti-inflammatory properties due to the presence of phenolic substances such as rosmarinate acid. Rosmarinic acid inhibits the production and release of pro-inflammatory mediators (Oliviu et al., 2017). Several studies have also indicated that polyphenols have an anti-inflammatory impact (Boşca et al., 2015).

Rosmarinic acid has been shown to inhibit complement activation both *in vivo* and *in vitro*. Rosmarinic acid inhibits complement activation by covalently reacting with the activated compliment component C3b, at the site of inflammation where complement activation is taking place, without the side effects of other drugs, such as glucocorticoids and anti-inflammatory drugs (Colica et al., 2018). Rosmarinic acid may be a potent inhibitor of the expression of the pro-inflammatory gene cyclooxygenase 2 (COX2). It is considered a risk factor for both HT29 cells of colon cancer and the non-malignant breast epithelial cell line MCF10A (Scheckel et al., 2008).

### 4.4 Chemical profile of hepatotoxic assay

Pyrrolizidine alkaloids (PAs) are common secondary plant metabolites that are widely distributed around the world, and about half of them have been reported to be hepatotoxic. PA is toxic in the liver through cytochrome P450 enzyme-catalyzed metabolic activation. Exposure to PA can cause liver damage such as liver sinusoidal obstruction syndrome (HSOS), cirrhosis, and cancer (Yokoyama et al., 2018; You et al., 2018). Lycopsamine is one of the pyrrolizidine alkaloid metabolites reported in the comfrey (Salehi et al., 2019). In this study, rosmarinic acid level influence the hepatotoxic level of comfrey beside the low level of lycopsamine (Table 2). Rosmarinic acid as phenolic metabolites in comfrey play a role for hepatoprotective profile (Touiss et al., 2021).

Rosmarinic acid is a diphenol metabolites found in many botanical drugs and spices and is considered a potential pharmaceutical natural product. Numerous reports have shown the hepatoprotective effect of rosmarinic acid through various mechanisms. These mechanisms include elimination or reduction of superoxide or peroxynitrite activity, reduction of indicators of liver toxicity such as aspartate aminotransferase, alanine aminotransferase, glutathione oxide, lipid peroxidation, and catalase, glutathione peroxidase, and super. Includes enzymatic activity associated with antioxidants such as oxidative dismutase. Rosmarinic acid also inhibited hepatic proliferation, TGFβ1, CTGF and αSMA expression in cultured HSCs. It reduced the degree of fibrosis and improved biochemical indicators and histopathological morphology. It also had a significant impact on inflammation and some inflammatory mediators (Elufioye and Habtemariam, 2019).

## 5 Conclusion

The betaine-urea-based NADES extraction was the better option for extracting rosmarinic acid and lycopsamine from comfrey (*Symphytum officinale* L.), with levels of 1.934 mg/g and 0.018 mg/g, respectively. The antioxidant, anti-inflammatory and hepatotoxic profile of betaine-urea NADES extraction was found to be considerable. Rosmarinic acid was shown to have antioxidant and anti-inflammatory chemical profile but also less hepatotoxic. Selective extraction towards rosmarinic acid over lycopsamine allows detoxification comfrey leaves, to further the investigation into the utilization of comfrey leaves as anti-inflammatory and antioxidant agent.

## Data Availability

The original contributions presented in the study are included in the article/supplementary material, further inquiries can be directed to the corresponding author.
